# Consistent pollen nutritional intake drives bumble bee (*Bombus impatiens*) colony growth and reproduction across different habitats

**DOI:** 10.1002/ece3.4115

**Published:** 2018-05-02

**Authors:** Anthony D. Vaudo, Liam M. Farrell, Harland M. Patch, Christina M. Grozinger, John F. Tooker

**Affiliations:** ^1^ Department of Entomology Center for Pollinator Research The Pennsylvania State University University Park Pennsylvania

**Keywords:** colony development, colony reproduction, foraging preferences, nutritional ecology, pollen quality, pollination

## Abstract

Foraging behavior is a critical adaptation by insects to obtain appropriate nutrients from the environment for development and fitness. Bumble bees (*Bombus* spp.) form annual colonies which must rapidly increase their worker populations to support rearing reproductive individuals before the end of the season. Therefore, colony growth and reproduction should be dependent on the quality and quantity of pollen resources in the surrounding landscape. Our previous research found that *B. impatiens* foraging preferences to different plant species were shaped by pollen protein:lipid nutritional ratios (P:L), with foragers preferring pollen species with a ~5:1 P:L ratio. In this study, we placed *B. impatiens* colonies in three different habitats (forest, forest edge, and valley) to determine whether pollen nutritional quality collected by the colonies differed between areas that may differ in resource abundance and diversity. We found that habitat did not influence the collected pollen nutritional quality, with colonies in all three habitats collecting pollen averaging a 4:1 P:L ratio. Furthermore, there was no difference in the nutritional quality of the pollen collected by colonies that successfully reared reproductives and those that did not. We found however, that “nutritional intake,” calculated as the colony‐level intake rate of nutrient quantities (protein, lipid, and sugar), was strongly related to colony growth and reproductive output. Therefore, we conclude that *B. impatiens* colony performance is a function of the abundance of nutritionally appropriate floral resources in the surrounding landscape. Because we did not comprehensively evaluate the nutrition provided by the plant communities in each habitat, it remains to be determined how *B. impatiens* polylectic foraging strategies helps them select among the available pollen nutritional landscape in a variety of plant communities to obtain a balance of key macronutrients.

## 1 INTRODUCTION

An appropriate quality and quantity of macronutrients are essential for development and reproduction of every organism (Behmer, 2009; Behmer & Joern, 2008). These nutritional needs are hypothesized to strongly influence foraging behavior, ensuring that an animal obtains required macronutrient (carbohydrate, protein, and lipid) intake from varied environments where the nutritional qualities of resources may differ (Jensen, Mayntz, Toft, Raubenheimer, & Simpson, 2011; Mayntz, Raubenheimer, Salomon, Toft, & Simpson, 2005; Raubenheimer, Mayntz, Simpson, & Tøft, 2007; Raubenheimer & Simpson, 1999; Simpson & Raubenheimer, 1993, 2012). Foraging bees obtain all their nutrients from floral pollen and nectar, and these floral resources vary among plant species in quality, quantity, and availability throughout space and time (Nicolson, Nepi, & Pacini, 2007; Petanidou, Kallimanis, Tzanopoulos, Sgardelis, & Pantis, 2008; Roulston & Cane, 2000; Willmer & Stone, 2004). Therefore, foraging bees must select among these resources to support their own homeostasis and reproduction, and provide nutrients for larvae confined to brood cells (Brodschneider & Crailsheim, 2010; Cane, 2016; Nicolson et al., 2007; Roulston & Cane, 2000). Bumble bees produce annual colonies, initiated by a single foundress queen, that ultimately comprise several hundred individuals (dependent on species) before reaching a “switching point” where the colony produces the next generation of reproductives (gynes and males) by the end of the growing season (Cnaani, Schmid‐Hempel, & Schmidt, 2002; Crone & Williams, 2016; Duchateau & Velthuis, 1988; Goulson, 2010; Williams, Regetz, & Kremen, 2012). Thus, these species must have continual access to quality floral resources for months to continuously grow the colony to a reproductive stage.

Global declines in bee populations, including bumble bees, have been linked to habitat degradation, including agricultural intensification, that reduces floral abundance and diversity (Biesmeijer et al., 2006; Goulson, Nicholls, Botías, & Rotheray, 2015) and the loss of key host‐plant species (Carvell et al., 2006). However, studies examining how variation in landscape influences bee health have focused on managed *Apis mellifera* honey bees (Otto, Roth, Carlson, & Smart, 2016; Requier et al., 2015; Smart, Pettis, Euliss, & Spivak, 2016; Smart, Pettis, Rice, Browning, & Spivak, 2016; Sponsler & Johnson, 2015). It is therefore not necessarily accurate to extrapolate the results of these studies to solitary bees or bumble bees, which have substantially smaller, annual colonies.

Only a handful of studies have monitored how landscape and floral resource availability influence dynamics of bumble bee colonies (Elliott, 2009b; Goulson, Hughes, Derwent, & Stout, 2002; Kämper et al., 2016; Lanterman & Goodell, 2017; Westphal, Steffan‐Dewenter, & Tscharntke, 2009; Williams et al., 2012). Colony growth appears to be strongly correlated with early‐season resource availability (Westphal et al., 2009; Williams et al., 2012), although growth has not been found to be a predictor of reproductive output (Crone & Williams, 2016; Goulson et al., 2002; Williams et al., 2012). In landscapes with higher diversity of floral resources, *B. terrestris* colonies grew more quickly and larger, yet did not differ in their ultimate output of reproductive individuals (Goulson et al., 2002). However, in *B. impatiens*, both colony growth and reproductive output were increased by floral diversity (Lanterman & Goodell, 2017). We expect that landscapes with differing degrees of floral resource diversity and abundance would lead to differences in pollen nutritional quality and quantity available, and ultimately differences in colony fitness. However, thus far the influence of landscape on colony‐level nutritional intake has not been explicitly examined.

Mounting evidence suggests that bumble bees (Hymenoptera: Apidae: *Bombus* spp.), especially *B. impatiens* and *B. terrestris*, show foraging preferences for plant species based on nutritional quality of pollen (Cardoza, Harris, & Grozinger, 2012; Hanley, Franco, Pichon, Darvill, & Goulson, 2008; Kitaoka & Nieh, 2008; Kriesell, Hilpert, & Leonhardt, 2016; Leonhardt & Blüthgen, 2011; Ruedenauer, Spaethe, & Leonhardt, 2016). Evolutionarily, this preference aligns with a goal of providing optimal resources for their brood, because suboptimal pollen quality can lead to reproductive deficit, egg cannibalism, and larval ejection (Génissel, Aupinel, Bressac, Tasei, & Chevrier, 2002; Tasei & Aupinel, 2008). In the laboratory, bumble bees prefer pollen diets with higher protein concentrations (Kitaoka & Nieh, 2008; Konzmann & Lunau, 2014; Ruedenauer, Spaethe, & Leonhardt, 2015; Ruedenauer et al., 2016), and these preferences extend to the field among plant species or within the same species (Cardoza et al., 2012; Hanley et al., 2008). Furthermore, bumble bee colonies will increase their foraging efforts to higher quality pollen (or nectar), or reduce foraging efforts to low‐quality pollen, even if no alternative is available (Dornhaus & Chittka, 2001, 2004; Kitaoka & Nieh, 2008). Our previous research revealed that *B. impatiens*, when collecting pollen for their colony in an enclosed outdoor foraging‐arena, preferred host‐plant species with pollen of high protein:lipid, or P:L ratios (~5:1 P:L, which was the maximum for the plant species in this study; Vaudo, Patch, Mortensen, Tooker, & Grozinger, 2016). Notably, foragers nearly ignored plant species offering the lowest P:L pollen (0.72:1 P:L), even when abundant pollen was available for collection (Vaudo, Patch et al., 2016). Additionally, in the laboratory in the absence of external floral cues and brood, *B. impatiens* maintained these P:L preferences among pollen from different species and exhibited preferences of 5:1–10:1 P:L from nutritionally modified pollens (Vaudo, Patch et al., 2016). Insect nutritional preferences exhibited in controlled settings may reflect the optimum that would be collected in the field. However, in different landscapes of floral abundance and diversity, bees may differ in their ability to meet their optimal nutritional needs and therefore affect colony fitness.

We tested whether bumble bees differ in their nutritional intake and colony growth in different habitats of a typical Pennsylvanian agricultural landscape—agricultural valley, field edge, and forest—that we expected to vary in floral diversity and abundance. We expected that field edges provide higher diversity of host‐plant species that should provide bumble bees nutritionally rich and season‐long forage availability and result in highest colony growth (Kammerer, Biddinger, Rajotte, & Mortensen, 2016). In contrast, forest summer floral resource diversity and abundance are lower, and agricultural land should have reduced floral diversity (punctuated by blooming of only a few crop species), both of which may result in lower colony growth and ability to obtain optimum nutrition (Goulson et al., 2002; Williams et al., 2012). We (1) determined whether nutritional quality of pollen collected by colonies varied among these three habitats; and (2) determined whether behavioral and nutritional factors (foraging rates, pollen quantity, and pollen quality) related to total nutritional intake rates influenced colony growth and reproduction. Overall, these data provide critical information integrating nutritional intake of bumble bees with colony behavioral dynamics, growth, and fitness.

## 2 METHODS

### 2.1 Site selection and bumble bee placement

**Figure 1:**
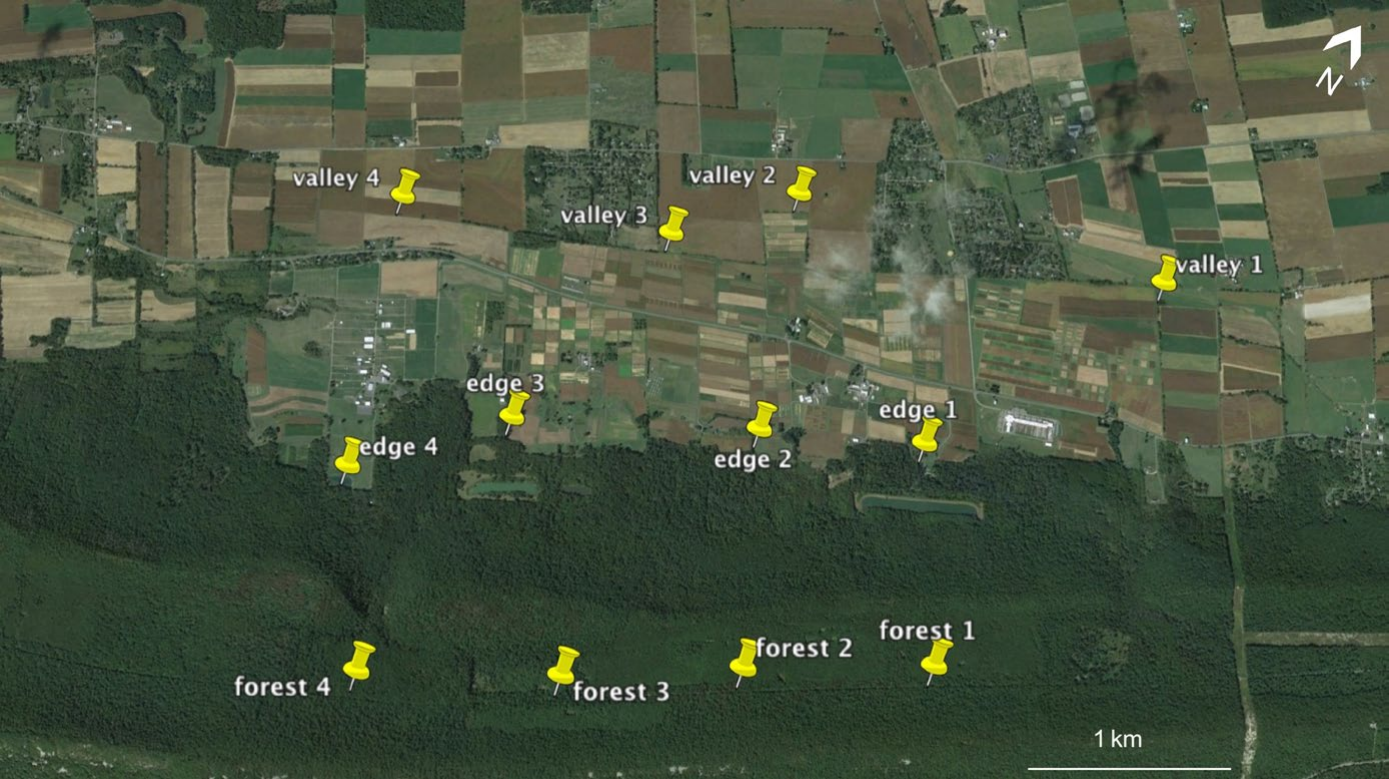
Field sites of the *Bombus impatiens* colonies that we deployed in central Pennsylvania. We placed two colonies at each site, facing opposite directions from each other (~E vs. W). The four sites in the Valley were along the field border of Penn State's Russell E. Larson Agricultural Research Center and were in a predominately agricultural landscape with two small residential neighborhoods. The four Edge sites were along the border of the research center and Rothrock State Forest. The Forest sites were placed ~5 m into the forest off Kepler Rd. in Rothrock State Forest. Photograph generated by Google Earth Pro v.7.1.5.1557

In an agricultural valley in central Pennsylvania (USA), we placed 24 bumble bee “research” colonies (Biobest Canada Ltd., Leamington, ON) in three typical habitats: (1) “Valley,” a comprising mainly agricultural land use, with some residential land; (2) “Edge,” the border between agriculture and forest habitats; and (3) “Forest,” a completely wooded habitat. We chose twelve sites (4 sites/habitat), arranged in a grid, such that each site was ~1 km apart from the others to minimize potential foraging range overlap between colonies (note that although no foraging range data are available for *B. impatiens*, we based the 1 km distance on estimates from other bumble bee species based on Darvill, Knight, & Goulson, 2004; Dramstad, 1996; Elliott, 2009a; Osborne et al., 1999; Walther‐Hellwig & Frankl, 2000; Figure 1). Valley and Edge habitats were on the land of Penn State's Russell E. Larson Agricultural Research Center, while the Forest habitats were in the adjacent section of Rothrock State Forest (Pennsylvania DCNR Bureau of Forestry research reference #SFRA‐1511; Figure 1). We placed two bumble bee colonies at each site with colony entrances facing opposite directions from each other. The colonies were elevated off the ground on cinder blocks and were secured to the blocks with twine. We tented heavy‐duty tarp above the colonies to protect them from direct sun and rain. At the start of the experiment, the colonies contained one queen and averaged 50 ± 2 workers and 73 ± 5 brood cells (mean ± SEM) and did not differ between sites (initial workers: *F*_11,23_ = 0.60, *p *=* *.79, initial brood cells: *F*_11,23_ = 0.96, *p *=* *.52). All colonies were deployed on 3 June 2015, and we removed the sugar water provided by the manufacturer.

### 2.2 Data collection

Each week of the study, on nonrainy days, we monitored the foraging rate each colony for 1 hr from ~0900 to 1600 (during bumble bee peak foraging time; Peat & Goulson, 2005; Stelzer & Chittka, 2010; Stelzer, Stanewsky, & Chittka, 2010), randomizing the order of sites observed each day and noting the time of day each observation occurred. We recorded the number of foragers returning to each colony with pollen loads (“pollen foraging rate”) and those without (“nonpollen foraging rate”; we could not visually determine whether they were truly nectar foragers). We collected corbiculate pollen loads from a maximum of five pollen foragers during the 1‐hr observation periods. As the pollen forager landed in the colony entrance, we held her by her mid‐ or hind leg with forceps and scraped the pollen loads off the corbiculae with soft forceps into 1.7 ml microcentrifuge vials. Pollen samples were placed on ice in the field and stored in −80°C until analysis. We analyzed each pollen load for its dry mass (mg) and recorded mg pollen collected per forager. We analyzed the pollen load protein, lipid, and sugar concentrations and protein:lipid ratio (P:L; Vaudo, Patch et al., 2016). Briefly, we analyzed the protein concentration of pollen using a modified Bradford assay and lipid and carbohydrate concentrations using an assay modified from Van Handel and Day (Van Handel & Day, 1988; see Vaudo, Patch et al., 2016 for protocol).

To measure the growth of colonies and obtain growth trajectories, each week, we visited the colonies once at night between 2300 and 0300 to weigh each colony. We weighed (g) the plastic box containing each colony using a Tree KHR3000 High Resolution Kitchen Scale (LW Measurements, LLC, Rohnert Park, CA). To obtain the biomass of the colony, we subtracted the average weight of five clean Biobest boxes (provided by Biobest) from the measured weight. To most accurately measure the biomass gained or lost completely in the field (and have a measurement robust against smaller fluctuations of biomass), we calculated the actual “maximum biomass” of each colony after the third week of the study. Because colonies had different initial masses, we also calculated “maximum biomass gain” of each colony as the initial mass subtracted from the “maximum biomass” after the third week of the study (such that all late instar larvae and pupae reared prior to delivery had emerged). We obtained daily maximum temperature data from the Natural Resources Conservation Service National Water and Climate Center weather station #2036.

We measured colony biomass until the colony failed to produce any more workers, completed rearing reproductives (males or gynes), or 7 August 2015, whichever came sooner. Any remaining pupae were allowed to emerge in the laboratory (note that by this date, all colonies were either completely senesced or surviving immature individuals were in the pupal stage). By creating a termination date, we were able to compare growth rates and reproduction within a structured timeframe. We counted the number of total cells as a measure of “lifetime population” (old cells are not reused to rear larvae). We counted the total number of reproductives (males or gynes) produced by each colony (only colony #18 produced gynes) and subsequently named any colony that produced reproductive individuals as reproductively “successful” for our analyses. We are confident we did not miscount reproductive output by colonies in the field as we monitored colonies frequently and reproductive individuals do not immediately exit the nest on emergence (Goulson, 2010).

### 2.3 Statistical analyses

All statistical analyses were conducted with JMP Pro 13.2.0 (SAS Institutes, Inc. 2016).

#### 2.3.1 Pollen nutrition

**Figure 2:**
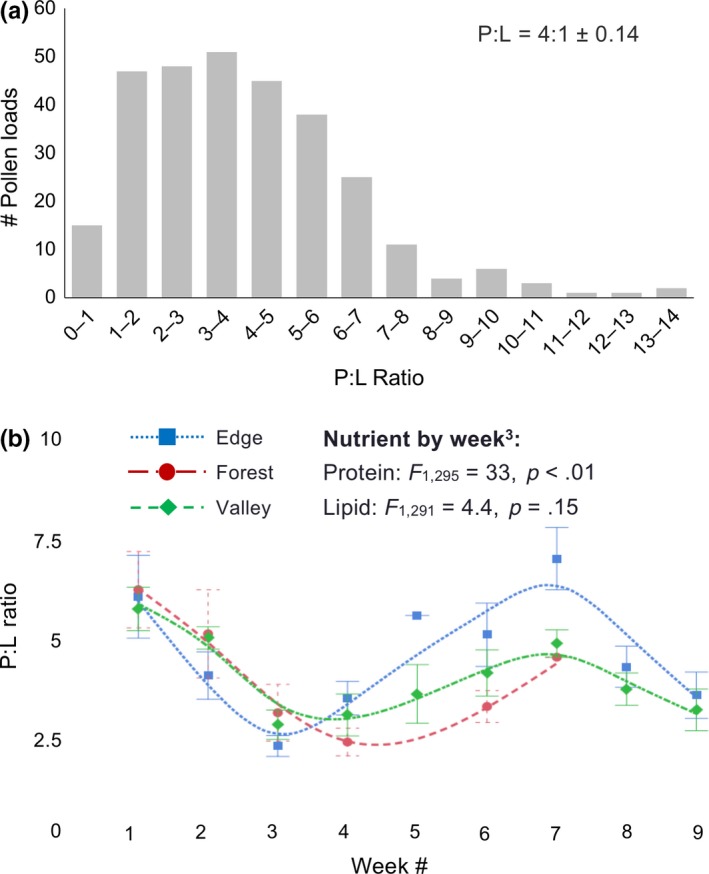
Distributions of protein:lipid ratios (P:L) from pollen collected from the corbiculae of individual *Bombus impatiens* foragers. (a) Distribution of P:L ratios of all individual forager pollen loads collected (*N = *297). Bars represent total numbers of corbiculate loads found within a given P:L range. (b) Mean P:L ratios (±*SE*) of pollen loads for each week of the study by habitat. P:L ratios did not differ between habitats each week yet exhibited a third‐order polynomial distribution over time. The smooth line was added to show the similar trends in pollen nutrition collected by colonies throughout the season in each habitat. Smoother lines are a cubic spline with lambda of 0.1 generated by JMP Pro 13.2.0. Note that protein concentrations of pollen collected by *B. impatiens* differed by week while lipid concentrations did not, suggesting that P:L ratios were driven by protein concentrations of pollen collected by bumble bees (see Section 2.1, Table 1)

We analyzed the distribution of all pollen loads by their protein, lipid, and sugar concentrations (μg nutrient/mg pollen) and protein:lipid (P:L) ratios. We determined whether pollen nutrition collected by colonies differed between habitats and over time using a nested mixed model analysis with “habitat” and “time (time + time^2^ + time^3^)” as fixed effects and “site” and “colony (nested within site)” as random effects. We tested time as a third‐order polynomial because of the observed trend in changes in pollen nutrition as the season progressed (see Figure 2). To determine whether pollen nutrition influenced reproductive success alone, we tested average pollen nutritional values between reproductively successful and unsuccessful colonies using ANOVA. Because we tested multiple factors with the same dataset, we used false detection rate analysis (FDR) to verify statistical significance of the models. To evaluate environmental factors that may influence pollen nutritional content, we analyzed the influence of daily temperature and time of day on the pollen nutrition using multiple regression analyses.

#### 2.3.2 Nutrition, behavior, growth, and reproduction

To determine the influence of habitat on colony foraging behavior, growth, and reproduction, we analyzed colony maximum biomass, maximum biomass gain, lifetime population, number of reproductives produced, and colony average nonpollen and pollen foraging rates with nested mixed model analysis with “habitat” and “site (nested within habitat)” as fixed effects, and “colony (nested within site)” as a random effect. Because we did observe differences in colonies by site that may not be driven by habitat alone, we kept site as a fixed effect. In a separate model, when analyzing the effects of habitat alone and excluding site as a factor, the results remained consistent (data not shown). Because we tested multiple factors with the same dataset, we used false detection rate analysis (FDR) to verify statistical significance of the model. We determined whether colonies differed in reproductive success (whether they produced at least one reproductive individual or not) between habitats with contingency analysis.

To determine whether the metrics of pollen nutritional quality were correlated to metrics of colony growth and reproduction, independent of habitat, we conducted a principal components analysis (PCA) with the following variables related to (1) pollen nutrition: colony average pollen protein, lipid, and sugar concentrations and P:L values; (2) foraging behavior and resource availability: colony average “pollen foraging rate” and “nonpollen foraging rate” and colony average pollen load mass collected per forager; (3) colony growth: lifetime population, maximum biomass, maximum biomass gain; and (4) reproduction: reproductive success (which we ordered 1 = “successful,” 0 = not reproductive) and total number of reproductive individuals produced. We used two principal components to determine amount of variance of the data we could explain. We used factor analysis that groups correlated variables to determine relationships between colony growth, behavior, and nutrition.

Because nutritional quality and quantity are integral to colony development, and the colony is the unit of reproduction for bumble bees, we developed a single metric to evaluate colony‐level nutritional intake that may account for different foraging strategies and environmental resource availability. “Nutritional intake” or “mg nutrient/hour” is calculated as colony average corbiculate pollen nutrient concentration multiplied by the colony average pollen mass collected per forager multiplied by the colony average pollen foraging rate (averaged across weeks):

nutritional intake=mg nutrienthr=μg nutrientmg pollen×mg pollencollected×no. of pollen foragershr

This metric provides a measure of quantity of nutrients foraged by each colony on an hourly basis or a rate of consumption of nutrients by the colony. Because colonies did not differ in average pollen protein, lipid, sugar concentrations, or P:L ratios (see Section 3 below), we measured intake of each nutrient separately to determine whether intake of any one nutrient was more predictive of colony health than others. To determine the relationship between nutritional intake and colony growth, we used regression analyses between nutritional intake and colony lifetime population and maximum biomass gain. To determine the relationship between nutritional intake and reproduction, we used *t* tests to evaluate the difference in intake between reproductively successful and unsuccessful colonies, and used regression analysis between nutritional intake and the number of reproductives produced by successful colonies.

## 3 RESULTS

### 3.1 Pollen nutrition

We collected pollen loads from the corbiculae of 301 *B. impatiens* foragers as they returned to their colonies and analyzed each load for nutritional quality (Table 1, Figure 2). Across all colonies, the P:L concentrations of pollen averaged 4:1 ± 0.14 (Table 1, Figure 2a). Protein, lipid, sugar, and P:L ratios did not differ between habitats (Table 1). Pollen nutrition did not differ statistically between reproductively successful or unsuccessful colonies (although there was a numerical trend for successful colonies to collect higher nutrient concentrations; Table 1). P:L ratios did vary throughout the season by week but not among habitats, indicating that bumble bee colonies were collecting similar quality resources in different habitats, contrary to our expectation that they would vary between habitats (Figure 2b; Table 1).

**Table 1:** Summary of pollen nutritional quality collected by *Bombus impatiens* colonies by habitat over time and reproductive success

	Pollen nutritional content											
	Protein (μg/mg pollen)			Lipid (μg/mg pollen)			Sugar (μg/mg pollen)			Protein:Lipid Ratio		
	*N*	Mean	*SE*	*N*	Mean	*SE*	*N*	Mean	*SE*	*N*	Mean	*SE*
*Habitat*												
Forest	45	181.86	15.46	45	57.65	4.34	45	380.21	18.73	45	3.72	0.43
Edge	100	192.74	9.39	99	53.42	1.40	99	395.62	13.45	99	4.00	0.26
Valley	156	213.31	6.50	153	55.15	1.54	154	362.94	10.21	153	4.27	0.17
	**H: ** *F*_2,291_ * = *6.7; *p = *.61			**H: ** *F*_2,287_ * = *1.2; *p = *.35			**H: ** *F*_2,288_ * = *1.3; *p = *.44			**H: ** *F*_1,287_ * = *0.59; *p = *.580		
	*T: *F*_1,291_ * = *33; *p *<* *.01			**T: ** *F*_1,287_ * = *4.4; *p = *.15			**T: ** *F*_1,288_ * = *1.9; *p = *.33			*T: *F*_1,287_ * = *42; *p *<* *.01		
*Reproduction*												
Unsuccessful	120	187.52	8.59	120	53.17	1.74	120	358.17	10.64	120	3.97	0.23
Successful	181	211.22	6.36	177	56.16	1.48	178	388.70	10.22	177	4.18	0.17
	*F*_1,20_ * = *0.5; *p *=* *.49			*F*_1,20_ * = *0.03; *p = *.90			*F*_1,20_ * = *2.7; *p = *.12			*F*_1,20_ * = *0.6; *p = *.45		

Note that nutritional values did not differ significantly between habitats and reproductive status; yet protein and P:L values did differ by week (see Figure 2b). For pollen nutrition, *F* and *p* values are provided for habitat (**H**) and time^3^ (**T)**. Significant differences (*p *<* *.05) denoted by asterisks (*) in pollen nutrition were verified with False Discovery Rate for multiple testing. For reproductive success, actual distributions of all pollen loads from corbiculae are shown, but ANOVA analyzed by colony average nutrient concentrations of collected pollen.

**Figure 3:**
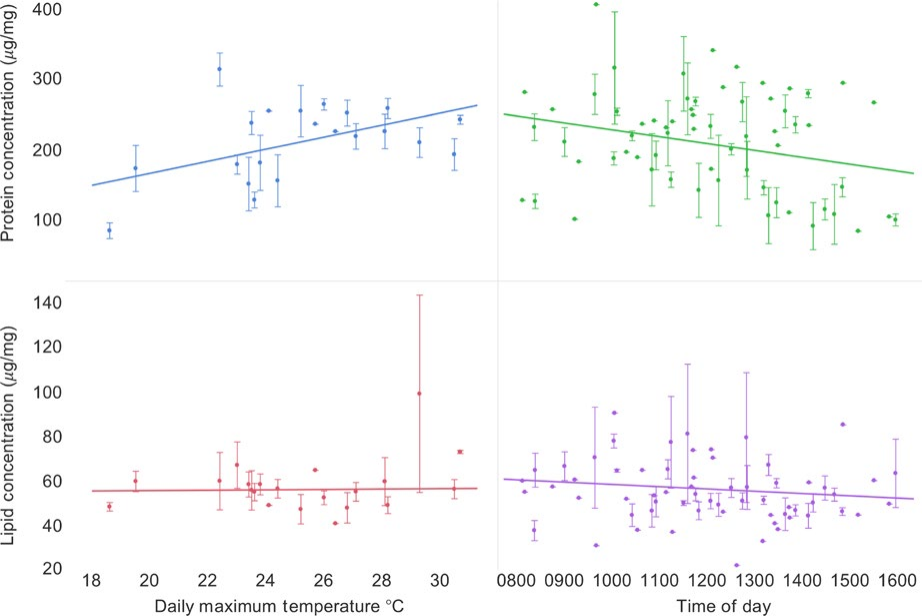
Regression of environmental variables on *Bombus impatiens* pollen nutrient concentrations. We conducted multiple regression on daily temperatures and time of day on pollen nutrient concentrations (shown as mean ± *SE*) collected by bumble bees. Protein concentrations of bumble bee‐collected pollen differed by maximum daily temperature and time of day (*F*_3,98_ *= *10.93, *p < *.01, *R* ^2^ *= *.26), while lipid concentrations did not (*F*_3,89_ *= *0.44, *R* ^2^ *= *.015) revealing environmental effects on bumble bee collection of pollen protein concentrations and consistent collection of pollen lipid concentrations

Notably, pollen protein concentration varied by week (showing a third‐order polynomial or cubic trend) whereas lipid concentration did not (Figure 2b; Table 1), suggesting host‐plant resource turnover, yet the bees potentially selected for consistent lipid concentrations. To evaluate environmental factors that may influence pollen nutritional content, we analyzed the influence of daily temperature and time of day on pollen protein and lipid collected. The interaction of daily temperature and time of day was significant for protein (protein concentrations increased as temperatures increased, but decreased as the day progressed; *F*_3,98_ = 10.93, *p < *.01, *R* ^2^ = .26, Figure 3), while neither time of day or daily temperature affected lipid concentration of pollen collected by bumble bees (*F*_3,89_ *= *0.44, *R* ^2^ *= *.015, Figure 3). Again, this suggests environmental influence on protein collection by bumble bees, while lipid concentrations remain consistent.

### 3.2 Nutrition, behavior, growth, and reproduction

Although there was only a trend toward statistical differences in colony reproductive success between habitats (χ^2^ *= *5.2, *p = *.07), there were differences in number of successful colonies (i.e., produced reproductive individuals; # successful: Forest: 1, Edge: 3, Valley: 5). Note that large mammals damaged the Forest colonies during the study and this undoubtedly contributed to their low rates of reproduction (all colonies were included in colony growth analyses because their development was uninhibited until the immediate termination of the colonies by the mammals). There were statistical differences among habitats in all categories of colony growth, reproduction, and foraging rates with Valley colonies consistently exhibiting higher values than Forest colonies (Table 2). Likewise, Valley colonies gained more mass, had higher lifetime populations, produced more reproductives, and had higher pollen foraging rates than Edge colonies (Table 2).

**Table 2:** Summary of growth, reproduction, and foraging rates of *B. impatiens* colonies (*N = *24)

	Growth and reproduction				Foraging rates (avg/colony)	
	Max biomass (g)	Biomass gain (g)	Lifetime population	No. of reproductives	Nonpollen foraging rate	Pollen foraging rate
	Mean ± *SE*	Mean ± *SE*	Mean ± *SE*	Mean ± *SE*	Mean ± *SE*	Mean ± *SE*
Habitat						
Forest	75.33 ± 9.57 b	9.51 ± 5.21 b	56.60 ± 8.36 b	0.11 ± 0.11 b	2.79 ± 1.12 b	6.97 ± 1.23 b
Edge	96.19 ± 11.65 ab	16.40 ± 6.27 b	93.60 ± 29.92 b	7.00 ± 4.37 b	4.10 ± 1.17 ab	7.17 ± 1.04 b
Valley	106.68 ± 29.29 a	48.15 ± 29.50 a	162.38 ± 26.32 a	21.63 ± 10.77 a	5.84 ± 1.42 a	12.70 ± 1.57 a
	**H: ** *F*_2,13_ * = *3.0	*H: *F*_2,13_ * = *9.5	*H: *F*_2,7_ * = *26.3	*H: *F*_2,13_ * = *17.3	**H: ** *F*_2,13_ * = *3.2	*H: *F*_2,13_ * = *10.0
	*p *=* *.09	*p = *.003	*p *<* *.001	*p *<* *.001	*p = *.07	*p = *.002
	*S: *F*_9,13_ = 9.9	*S: *F*_9,13_ * = *15.5	*S: *F*_8,7_ * = *8.2	*S: *F*_9,13_ * = *13.1	*S: *F*_9,13_ * = *4.2	*S: *F*_9,13_ * = *2.79
	*p *<* *.001	*p *<* *.001	*p = *.006	*p *<* *.001	*p = *.01	*p = *.046

*F* and *p* values are provided for the effects of habitat (**H**) and site nested within habitat (**S**). Means between habitats within each category denoted with different letters are significantly different (*p *<* *.05). Significant differences (*p *<* *.05) denoted by asterisks (*) in colony dynamics between habitats and sites were verified with False Discovery Rate for multiple testing. Note the trend that Valley colonies outperformed Forest colonies in all tests, but colony dynamics also always revealed significant site‐specific differences (see Sections 2.1 and 2.2).

**Figure 4:**
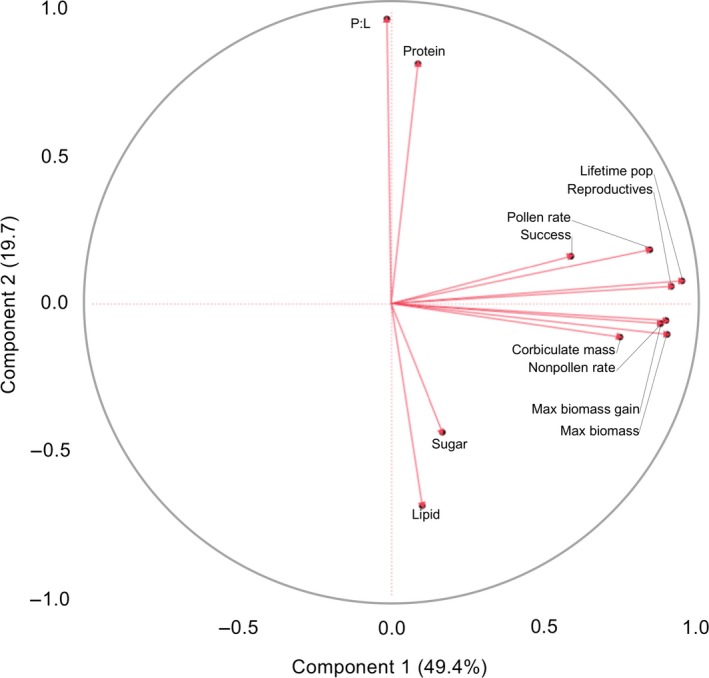
Principal component analysis for season‐long colony development, behavior, and nutrition of *Bombus impatiens*. PCA grouped factors associated with resource abundance and foraging behavior (pollen and nonpollen foraging rates and corbiculate pollen mass) with colony growth and reproduction (maximum biomass and biomass gain, lifetime population, reproductive success, and number of reproductives). Pollen nutrition was not correlated to colony dynamics because it did not differ between colonies (see Section 2.1) and was assigned to a second factor where lipid and sugar concentrations were negatively correlated to protein concentration and P:L ratios

Independent of habitat, we determined what nutritional and behavioral factors correlated with colony growth and reproduction using PCA. The analysis divided the data into two factors accounting for 69.1% of the variance (Figure 4). Factor 1 (49.4% of the variance) included the positively correlated variables related to behavior, growth, and reproduction: average pollen foraging rate, average nonpollen foraging rate, corbiculae pollen mass, colony maximum biomass gain, colony maximum biomass, lifetime population, colony success, and number of reproductives produced by each colony. Factor 2 (19.7% of the variance) included pollen nutritional variables with pollen protein and P:L negatively correlated with pollen sugar and lipid concentrations (Figure 4).

**Figure 5:**
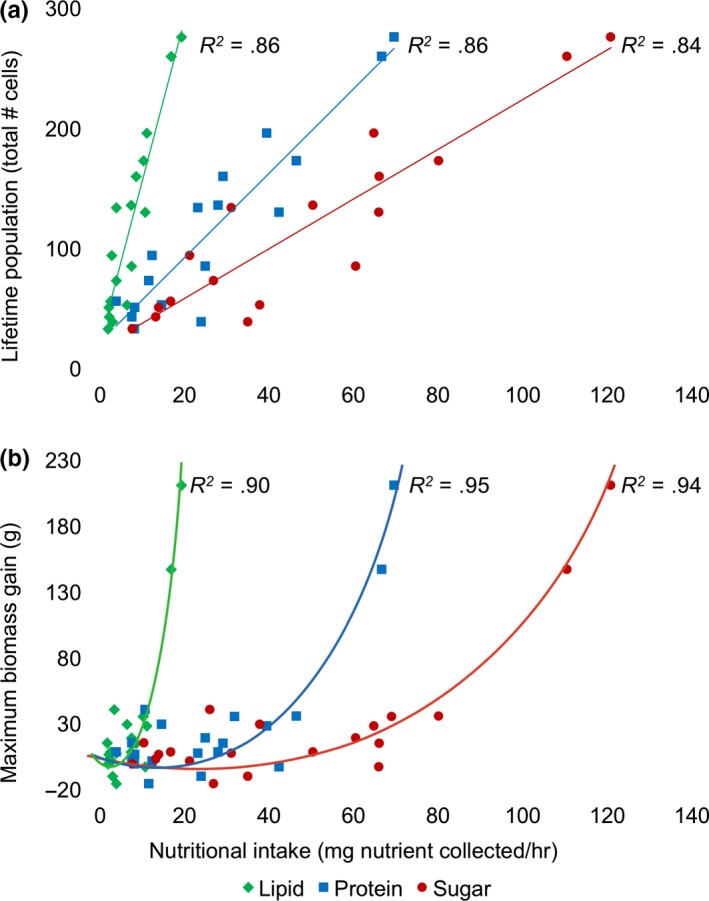
Colony‐level nutritional intake was highly correlated to colony (a) lifetime population and (b) maximum biomass gain. Nutritional intake was calculated as colony‐level average mg lipid, protein, and sugar foraged per hour (see Section 2 for formula)

**Figure 6:**
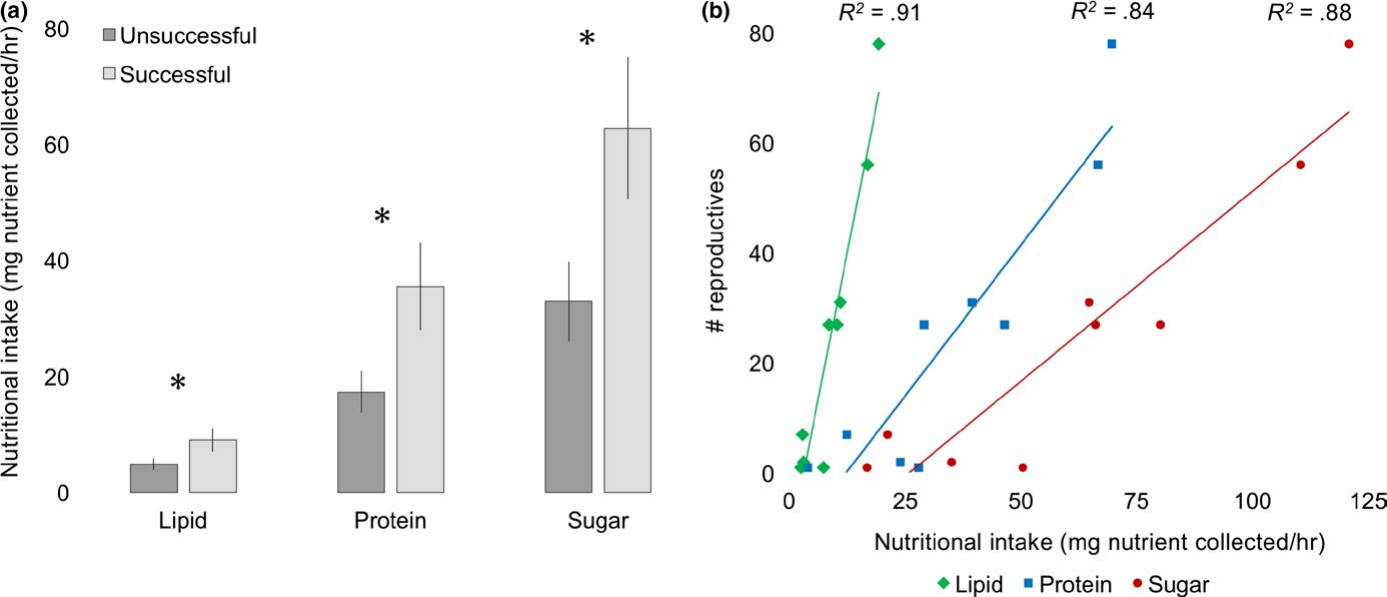
Nutritional intake positively affected colony reproduction. (a) Nutritional intake (mean ± *SE*) for lipid, protein, and sugar was higher in colonies that were reproductively “successful,” producing at least one reproductive individual. Asterisks represent statistical difference at *p *<* *.05. (b) Nutritional intake of lipid, protein, and sugar was linearly correlated to the number of reproductive individuals produced by “successful” colonies. Nutritional intake was calculated as colony‐level average mg lipid, protein, and sugar foraged per hour (see Section 2 for formula)

“Nutritional intake” for each macronutrient was significantly correlated to total colony population (protein intake: *F*_1,15_ *= *92.3, *p < *.01, *R* ^2^ *= *.86, lipid intake: *F*_1,15_ *= *90.5, *p < *.01, *R* ^2^ *= *.86, carbohydrate intake: *F*_1,17_ *= *78.6, *p < *.01, *R* ^2^ *= *.84; Figure 5a) and colony maximum biomass gain (protein intake: *F*_1,17_ *= *62.8, *p < *.01, *R* ^2^ *= *.88, lipid intake: *F*_1,17_ *= *97.3, *p < *.01, *R* ^2^ **= .92, carbohydrate intake: *F*_1,17_ *= *90.4, *p < *.01, *R* ^2^ *= *.91; Figure 5b). Nutritional intake was also significantly higher in colonies that produced reproductive individuals than those that did not (protein intake: *t*_19_ *= *2.3, *p *=* *.02; lipid: *t*_19_ *= *0.03, *p = *.01, sugar: *t*_22_ *= *2.2, *p = *.02; Figure 6a). Finally, colony‐level nutritional intake of all the macronutrients found in corbiculate pollen was strongly positively correlated to the number of total reproductive individuals produced by a successful colony (protein intake: *F*_2,6_ *= *26.1, *p < *.01, *R* ^2^ *= *.90, lipid intake: *F*_1,8_ = 56.1, *p < *.01, *R* ^2^ *= *.95, carbohydrate intake: *F*_2,6_ = 43.7, *p < *.01, *R* ^2^ *= *.94; Figure 6b).

## 4 DISCUSSION

Our study is the first to test the nutritional quality of pollen collected by *Bombus impatiens* over time while nesting in different habitats. Further, the study appears to be the first to incorporate the nutritional value of pollen into predictions of colony fitness. Our approach began by evaluating the nutritional quality of pollen collected, then we determined what environmental, behavioral, and colony factors lead to colony growth, and finally, we created a metric of nutritional intake to integrate these factors and predict how the rate of nutrient consumption affects colony growth dynamics and reproductive output.

### 4.1 Pollen nutrition

Protein, lipid, carbohydrate, and P:L values of pollen collected by colonies did not differ between habitats (Table 1), contrary to our expectation that floral diversity would differ between habitats (increasing at the field edge habitat) and lead to differences among the nutrients collected by colonies. The pollen collected in each habitat averaged a ~4:1 P:L ratio. This is similar finding to our previous research wherein semi‐field and laboratory choice assays *B. impatiens* preferred pollen with a ratio of 5:1 (Vaudo, Patch et al., 2016). In the current study, the distribution of pollen across the colonies and weeks was both above and below 5:1 (Figure 2a), indicating substantial variation in the P:L ratios of available pollen in the landscape. Among the diversity of pollen nutrients available throughout the landscape, bumble bees in each habitat were still able to converge on an average P:L ratio that resembled our predictions from our controlled experiments (Vaudo, Patch et al., 2016).

These results do not definitively answer the question of whether *B. impatiens* selectively foraged for a 4:1 P:L ratio or if they passively collected what was available in the landscape, which would require that the floral plant communities in these different landscapes all exhibited an average of 4:1 P:L ratios. Our hypothesis that bumble bees selectively forage for nutrition is supported by choice assays and empirical surveys. When fed modified pollen or synthetic diets, *B. impatiens* preferred and survived best on 5:1–10:1 P:L diets (Vaudo, Patch et al., 2016; Vaudo, Stabler et al., 2016). But high pollen P:L ratios such as 10:1 may be rare or in low abundance in the field (see the following references for pollen nutritional concentrations: Roulston & Cane, 2000; Roulston, Cane, & Buchmann, 2000; note that pollen P:L ratios so far have only been calculated in Vaudo, Patch et al., 2016). In an empirical survey of the nutritional content of pollen of 68 bee‐pollinated plant species, 52 had P:L ratios below 4:1 P:L (unpublished data). This suggests that pollen P:L ratios greater than 4:1 pollen may be relatively uncommon in the landscape. Several studies have suggested that bumble bees do selectively forage in the field: They tend to visit plant species with higher pollen protein content (Hanley et al., 2008) and exhibit foraging behavior to obtain quality (nutrient content or abundant) pollen including traveling further for higher quality resources (Jha & Kremen, 2013; Pope & Jha, 2018; Ruedenauer et al., 2016). Additionally, when foraging in the same landscapes, bumble bees collect pollen higher in protein content than honey bee colonies (Leonhardt & Blüthgen, 2011). Nevertheless, it is not possible to definitely test the hypothesis that *B. impatiens* selectively forages for particular P:L ratios in floral communities in the field until one assesses the actual distribution of pollen nutrition and abundance across all host‐plant species in the landscape and compares it against the nutritional content of pollen brought back to colonies.

Protein‐to‐lipid ratios of collected pollen varied over the season, yet these were driven by variation in protein content while lipid concentrations of corbiculate loads remained surprisingly consistent (5.5 ± 1.1% lipid; Figure 2b). Similarly, the protein content of collected pollen varied with the temperature and time of day at which foragers were foraging while lipid content did not (Figure 3). These results are similar to our previous findings that *B. impatiens* collected more preferred pollen earlier in the day and then moved to lower quality resources (Vaudo, Patch, Mortensen, Grozinger, & Tooker, 2014; Vaudo, Patch et al., 2016). The trend for bees to collect higher protein content pollen on warmer days is also consistent with the finding that bumble bees are more likely to collect pollen in dry and warm conditions (Peat & Goulson, 2005) and perhaps are more selective for pollen quality under such conditions. Therefore, although pollen resources available to *B. impatiens* appear environmentally driven, through seasonal and daily phenology, their consistent collection of pollen lipid concentration is striking. As noted above, whether this consistency results from bumble bees actively collecting from quality (high protein content, low lipid content, or abundant) patches of particular floral resources (Jha & Kremen, 2013; Ruedenauer et al., 2016), or simply due to average lipid content from random collection, remains to be determined.

### 4.2 Nutrition, behavior, growth, and reproduction

Although nutritional quality of pollen across all colonies and habitats did not differ, there were still differences in levels of colony growth between habitats. In particular, Valley colonies outperformed Edge and Forest colonies (Table 2), contrary to our expectations that colonies nesting along the field edge would have season‐long diverse pollen resources leading to increased colony growth and reproductive output. This suggests that Valley (agricultural and residential land) colonies may have had easier access to more preferred pollen patches, increasing foraging rates and worker populations in the agricultural habitat, which is consistent with other studies (Requier et al., 2015; Sponsler & Johnson, 2015) indicating that agricultural landscapes—which themselves contain a great deal of edge habitat and weedy plants—can provide abundant and diverse resources for bees. Additionally, proximity to forested land, and perhaps the lower floral diversity in this region, may have led to lower colony growth and reproduction in Forest and Edge habitats (Lanterman & Goodell, 2017).

We attempted to separate colonies such that they may not overlap in foraging ranges or resources, yet they may have collected from similar patches of flowers. Foragers from colonies at different sites may have foraged further distances to reach these patches however (Pope & Jha, 2018), perhaps causing energetic stress and differences in colony growth. Indeed, there were significant differences between sites in all categories of growth, reproduction, and foraging, and therefore, broad classification of habitats alone did account for differences in colony health (Table 2; see “Case Study” in Supporting Information and Figure S1). For instance, Valley 2 colonies were placed in an agricultural area where hedgerows, neighborhoods, and flowering crops likely provided more consistent floral resource availability and diversity leading to increased health (Alaux et al., 2016; Goulson et al., 2002). Edge 4 colonies were placed in the only perceivable area with wildflower abundance along the forest edge, possibly explaining the success of these colonies compared to other Edge sites. In contrast, Valley 4 colonies were placed in wheat and corn fields without any obvious wildflower habitat. Therefore, assessing floral resources in proximity to colonies (Williams et al., 2012) would likely be more predictive of resource diversity, abundance, and quality than assumptions based on general habitat type (Lonsdorf et al., 2009; Requier et al., 2015).

We therefore chose to determine what nutritional and behavioral factors were most predictive of colony reproductive growth and fitness independent of habitat using PCA (Figure 4). Pollen nutrition was not correlated to any measure of colony growth, again because there were no differences in colony‐level nutritional quality of pollen. Colony foraging rates, corbiculate pollen mass, colony biomass, and total population were all correlated with colony reproductive success (Figure 4), supporting the model that (1) nutritional quantity—likely determined by floral abundance—is critical for colony development (Williams et al., 2012) and (2) increasing colony size is critical for colonies to switch to producing new reproductives (Westphal et al., 2009).

We developed a “nutritional intake” metric that integrates nutrition and behavior to evaluate the colony‐level rate of nutrient consumption how this influences growth and reproduction. We evaluated nutritional intake separately for protein, lipid, and carbohydrates to determine whether any one nutrient was more predictive of colony growth than total nutrients (and to be used as a tool for evaluating colony growth in future studies). For all three macronutrients, colonies exhibiting higher nutritional intake were able to outgrow other colonies and produce higher numbers of reproductives (Figures 5, 6). Colony #18, which was the only colony to produce gynes (all the other reproductive colonies produced males), had the highest nutritional intake among all colonies for protein, lipid, and sugar exemplifying the use of this metric.

These results suggest a positive feedback loop between environmental availability of resources (nutritional quantity) and pollen foraging rates and capacity (Dornhaus, Brockmann, & Chittka, 2003; Dornhaus & Chittka, 2001, 2004, 2005; Hendriksma & Shafir, 2016; Kitaoka & Nieh, 2008): Better resources lead to higher worker populations and increased foraging (Amsalem, Grozinger, Padilla, & Hefetz, 2015), which led to higher colony growth and reproduction. Thus, *B. impatiens* foragers appear to have collected pollen that at least met the minimum nutritional requirements of developing larvae in all cases, but the *quantity* of these pollen resources influenced the colony growth rate and ability to transition to their reproductive phase (Goulson et al., 2002; Jha & Kremen, 2013; Kämper et al., 2016; Kriesell et al., 2016; Lanterman & Goodell, 2017; Ruedenauer et al., 2016; Vaudo, Patch et al., 2016; Westphal et al., 2009; Williams et al., 2012). Further, our results suggest that *B. impatiens* can exhibit plasticity in colony growth and behavioral dynamics that accommodate nesting‐site specific differences in floral resources, allowing colonies to reach reproductive maturity despite their immediate resource base (Supporting Information; Figure S1). Further studies are needed to (1) determine the interaction of nutritional quality and quantity that yields maximum colony growth and (2) determine the interacting environmental and nutritional factors that contribute colony reproductive (male to gyne ratio) output.

## 5 CONCLUSIONS

In this study, we expected that differences in habitat would lead to differences in colony performance and success, which would be associated with variation in nutritional intake quantity or quality. Our data indicate that *B. impatiens* collected similar pollen nutritional resources (in terms of the macronutrient ratios) in all three habitats where we placed colonies, although time of the season influenced these variables (Kämper et al., 2016; Kriesell et al., 2016). However, there were clear differences in colony foraging rates, growth, and reproduction in these different habitats. Furthermore, we found that colony development strongly correlated with foraging resource quality and availability, as reflected by foraging rates, the amount of pollen collected, and the total macronutrient quantity the foragers brought in to the colony. Thus, poor performance was associated with reduced collection of nutritionally suitable floral resources from the habitat. This reduction in foraging effort may have resulted from fewer floral resources in the landscape or workers having to travel farther to find appropriate resources, both of which could have reduced the foraging force (Dornhaus & Chittka, 2001, 2004; Génissel et al., 2002; Kitaoka & Nieh, 2008; Pope & Jha, 2018) and nutritional intake of colonies. Future studies should analyze the nutritional quality of both bee‐collected pollen and the local plant communities to determine how bumble bees—and indeed, other bee species—selectively versus opportunistically forage among a variety of pollen nutritional landscapes to meet their nutritional requirements.

## CONFLICT OF INTEREST

None declared.

## AUTHOR CONTRIBUTIONS

ADV, HMP, CMG, and JFT conceived the ideas and designed methodology; ADV and LMF collected the data; ADV and LMF analyzed the data; ADV led the writing of the manuscript. All authors contributed critically to the drafts and gave final approval for publication.
