# Supplementary figures and images for: Consistent pollen nutritional intake drives bumble bee (Bombus impatiens) colony growth and reproduction across different habitats
Source: Ecol Evol. 2018 May 2;8(11):5765–76. doi: 10.1002/ece3.4115 (PMC6010792; doi:10.1002/ece3.4115)

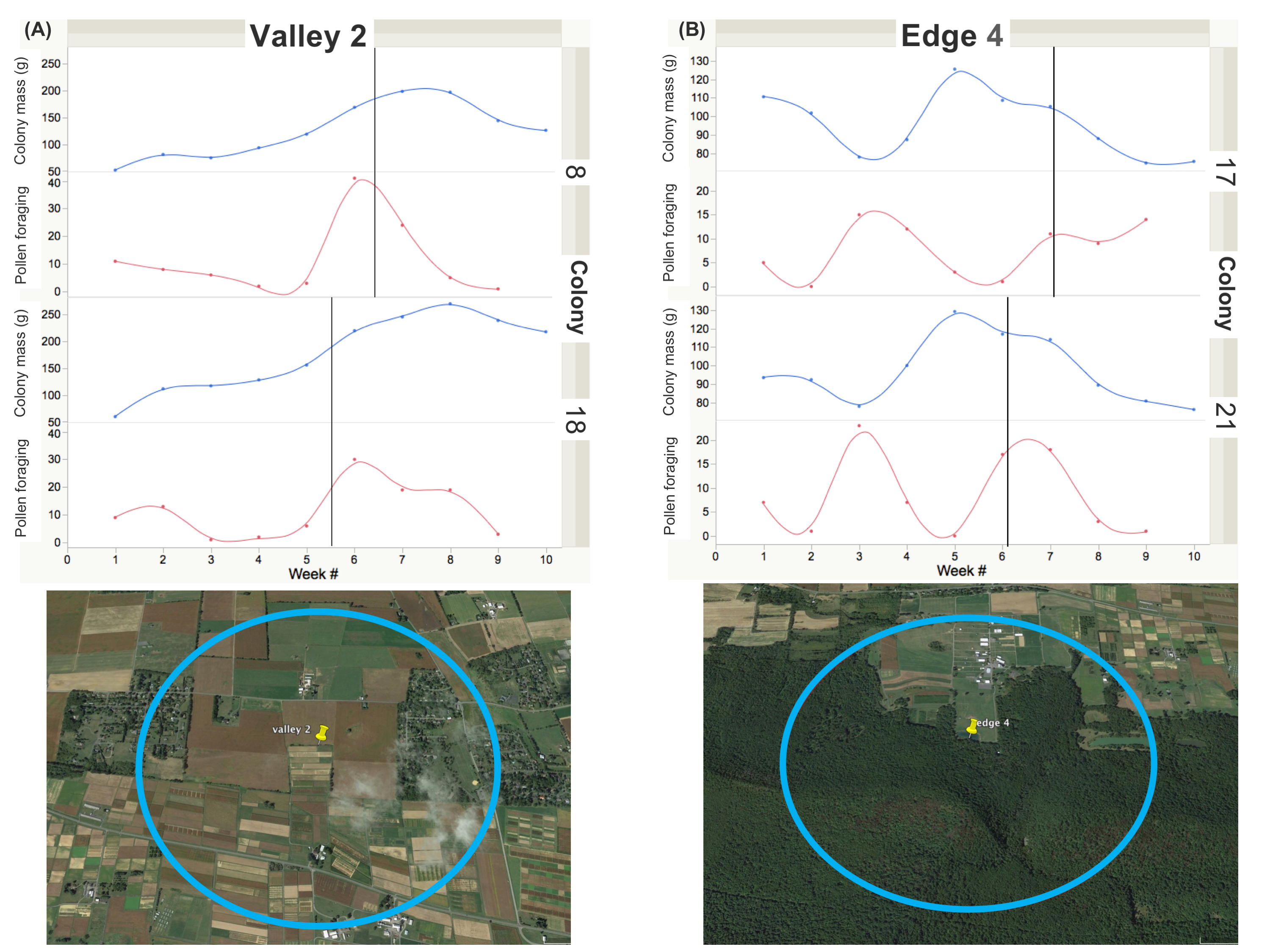

Supplement: Supplementary file 1 [file ECE3-8-5765-s001.tiff]
