# Supplementary material for: Consistent pollen nutritional intake drives bumble bee (Bombus impatiens) colony growth and reproduction across different habitats
Source: Ecol Evol. 2018 May 2;8(11):5765–76. doi: 10.1002/ece3.4115 (PMC6010792; doi:10.1002/ece3.4115)
Supplement: Supplementary file 2 [file ECE3-8-5765-s002.docx]

**Supporting Information**

Case study of two ‘best’ field sites:

To inspect different trends in colony dynamics noticed in the field, we graphically examined the similarities and differences of growth trajectories and pollen foraging rates between the four most reproductively successful colonies, located at sites Valley 2 and Edge 4. We plotted the weekly biomass trajectories of each colony and weekly pollen foraging rates. We also overlaid the estimated occurrence of the switching point of each colony, or time male or gyne eggs would have been laid, back-calculated from the time of the emergence of adult males and gynes (Cnaani et al. 2002).

The sites Valley 2 (colonies 8 and 18) and Edge 4 (colonies 17 and 21; Figure 1) had the highest reproductive output (Colony 8: 56 males; Colony 18: 60 males, 18 gynes; Colony 17: 27 males; Colony 21: 27 males). The colonies within each of these two sites had parallel growth trajectories, but the growth trajectories differed substantially between sites. Importantly, pollen-foraging rates also were similar between colonies within sites (Figure S1). Valley 2 colonies experienced constant colony growth, with rather stable pollen-foraging rates. Pollen foraging rates spiked between weeks six and seven, and this spike in pollen foraging correlated with the colony switch to rearing reproductives (Figure S1a). At Edge 4, however, there was an initial pollen-foraging spike at week three that corresponded to a loss in colony weight, and then subsequent weight gain for the following two weeks. There was a second increase in pollen-foraging rates at Edge 4, again corresponding with the colonies’ switching points (Figure S1b).

These data indicate clear environmental effects on colony growth and reproductive outcome, but demonstrate that colonies can still grow and reproduce under different conditions. What remained consistent between all colonies was pollen-foraging increases at the time of the colonies’ switching points (Figure S1). It remains to be determined if the rearing of reproductive individuals caused the spike in pollen-foraging rates at both sites, or if the spike in foraging rates triggered the rearing of reproductive individuals (Dornhaus et al. 2003; Dornhaus and Chittka 2005; Kitaoka and Nieh 2008). In the case of Edge 4 colonies, it appears that pollen-foraging efforts changed in response to colony status because pollen foraging rates increased following colony biomass loss. This foraging response could be that *B. impatiens* have a reserve of unspecialized (pollen or nectar) foragers that could change roles when in need (Hagbery and Nieh 2012).

**Figure S1. Colonies within sites showed similarities in behavior and growth trajectories, but varied across sites.** (A) Valley 2 (colonies 8 and 18) and (B) Edge 4 (colonies 17 and 21), the two sites that produced in both colonies the highest total number of reproductives. Graphs show colony-growth trajectories and pollen-foraging rates by week. Maps show local habitat of each site with 1km approximate foraging radius surrounding each colony (note no foraging range data are available for *B. impatiens*, for other bumble bee species, see Dramstad 1995, Osborne et al. 1999, Walther-Hellwig and Frankl 2000, Darvill et al. 2004, Elliott 2009a). Vertical lines on each graph represent hypothesized switching point to producing reproductive individuals (back calculated from emergence of reproductives), showing the synchrony between the switching points and pollen-foraging spikes.
